# Parental Stress, Parent-Child Relationship, and Child Wellbeing: A National Study of Family Life After COVID-19 Pandemic

**DOI:** 10.3390/bs15101423

**Published:** 2025-10-20

**Authors:** Vanessa Rodriguez, Jessica Cottrell, Fanli Jia

**Affiliations:** 1 Department of Physician Assistant, Seton Hall University, Nutley, NJ 07110, USA; 2 Department of Biological Sciences, Seton Hall University, South Orange, NJ 07079, USA; 3 Department of Psychology, Seton Hall University, South Orange, NJ 07079, USA

**Keywords:** parent–child relationship, COVID-19, stress, anxiety, daily activity routine, mental and physical well-being

## Abstract

The COVID-19 pandemic introduced profound and rapid disruptions to family life, particularly affecting parents through altered routines, economic instability, and reduced access to childcare and social support. This study investigates the cascading effects of these stressors on parent–child relationships and their influence on children’s mental and physical well-being. Drawing on a U.S. based national sample, findings reveal that parental daily routine disruptions were significantly associated with adverse child outcomes. The quality of the parent–child relationship emerged as a central mediating factor, fully mediating mental health outcomes and partially mediating physical health impacts. While parental anxiety was a factor, its mediating impact was less pronounced. After peaking in 2020, modest improvements were observed by the end of 2022, yet residual stress and incomplete recovery highlight the enduring psychological impact of the pandemic. These results underscore the importance of strengthening parent–child relationships and alleviating parental burdens during public health crises. Effective policy responses should integrate mental health resources, flexible employment options, and parenting-focused programs that reinforce family stability and developmental outcomes.

## 1. Introduction

The COVID-19 pandemic brought about an unprecedented global disruption that profoundly reshaped the daily lives of families. Among those most affected were parents, who experienced sudden shifts in responsibilities, including remote work, home schooling, loss of childcare, and economic instability. These rapid changes placed immense strain on family dynamics, particularly the routines and mental health of parents (Aznar et al., 2021; Billing et al., 2023). As a result, parental stress increased significantly (Hiraoka & Tomoda, 2020), influenced by job loss, financial insecurity, and social isolation (Lee et al., 2021; Chung et al., 2022b; Yuan et al., 2020). These stressors compounded over time, leading to notable changes in parenting behaviors and, more importantly, the quality of the parent–child relationship (Coyne et al., 2020; Crawford, 2021).

Previous research has shown that parental stress and anxiety can disrupt parenting practices, often resulting in more punitive or less emotionally available interactions with children (Jackson & Choi, 2018; Wilson & Durbin, 2010). Such changes in parental engagement are associated with increased behavioral problems and decreased emotional well-being in children (Crnic et al., 2005; S. H. Goodman et al., 2011). Moreover, studies emphasize that the parent–child relationship serves in the transmission of stress from parent to child, influencing outcomes related to the child’s mental, behavioral, and physical health (Ackard et al., 2006; Deater-Deckard & Scarr, 1996).

In addition to the profound challenges faced by parents, children themselves experienced direct and significant disruptions to their daily lives during the pandemic. School closures and the abrupt transition to remote learning led to academic setbacks, reduced access to educational resources, and widening achievement gaps (Kuhfeld et al., 2020; UNESCO, 2021). Social distancing measures limited opportunities for peer interaction, contributing to increased feelings of loneliness, social withdrawal, and difficulties in maintaining friendships (Loades et al., 2020). Moreover, many children demonstrated heightened anxiety, depressive symptoms, sleep disturbances, and behavioral difficulties as routines and support systems were destabilized (Singh et al., 2021a; Lehmann et al., 2022). These impacts highlight how children, independent of parental stress, were uniquely vulnerable to the effects of the pandemic, underscoring the importance of examining both child-specific and relational outcomes in understanding family well-being.

Understanding how these cascading effects unfolded during the pandemic is essential for identifying the mechanisms through which both parents and children were individually affected, as well as how their relationship was shaped. This study investigates how pandemic-related social restrictions affected parent–child relationships and subsequently influenced children’s mental and physical well-being. Specifically, it explores the extent to which parental daily routines and anxiety levels during the pandemic shaped parenting engagement and communication, and how these factors mediated children’s developmental outcomes.

By drawing on a U.S.-based national sample and examining changes from the self-reported worst time of the pandemic through the end of 2022, the current research aims to identify both the persistent consequences of the pandemic and the resilience of families over time. The findings are intended to inform future interventions and policies that support family well-being during public health emergencies or similar societal disruptions.

### 1.1. Parent Daily Routine

Daily life of citizens shifted during the COVID-19 pandemic. Parents around the world were not spared from the significant transition the pandemic caused in daily routines (Billing et al., 2023). The closing of schools, child-care establishments, parks, and playgrounds altered parental responsibilities (Aznar et al., 2021). Parents nevertheless worked from home while struggling to home school their children. Government restrictions during the pandemic disrupted parents’ abilities to obtain household essentials (Chung et al., 2022b). Lack of employment interrupted the standard of living for many families (Chung et al., 2022b). Economic insecurity worldwide pressured parents to raise their children through uncertain conditions with constrained means. The mental health concerns of parents inevitably increased during the COVID-19 pandemic (Yuan et al., 2020).

### 1.2. Parent Stress

Any mass trauma on a global scale such as the worldwide COVID-19 pandemic can result in a multitude of various stressful outcomes affecting finances, physical health, mental health, and social well-being. Cumulatively, these consequences affect daily life and can snowball stress from parenting. Social distance restrictions decreased measures that support households through parenting stress. Limiting access to families, friends, clubs, church groups, and other community gatherings that support parents limits the outlets available to release stress incurred from daily routines in parenting. Parents shouldered all-consuming overlapping responsibilities during the pandemic (Coyne et al., 2020). Moreover, parental stress continued to evolve from a lack of interpersonal connection outside of the home (Crawford, 2021). Pandemic restrictions and concerns about illness limited healthy coping mechanisms such as outside interactions and exercise routines, which contributed to sleep difficulties and substance abuse (Singh et al., 2021a). 

### 1.3. Parent–Child Relationship

Parents who exhibit greater amounts of stress are more likely to establish parenting styles that are harsh or punitive (Jackson & Choi, 2018; Chung et al., 2022a). Stress among parents can also negatively impact the parent–child relationship (Cummings et al., 2005). Parents with higher stress levels have been correlated with increased conflict in their relationships with their children (Crnic et al., 2005). Similarly, parents experiencing elevated stress exhibit decreased levels of communication with their children (Ponnet et al., 2013), while those presenting with feelings of depression are less warm toward their children (Wilson & Durbin, 2010). Increased parental stress can lead to a disruption of effective parenting and less time spent with children (Bronte-Tinkew et al., 2010). The struggle within households to manage mental health influenced parenting styles (Low & Mounts, 2022).

### 1.4. Parent Anxiety/Stress and Child Mental Health and Wellbeing

Parent mental health has been linked to child mental and physical health. Studies have demonstrated the correlation between declining parental mental health and poorer psychological and behavioral outcomes for their children (Barroso et al., 2018; S. H. Goodman et al., 2011). Young children have displayed more behavioral issues after interacting with their stressed parents (Anthony et al., 2005). In some cases, parents experiencing stress transfer these struggles to their children through the parent–child relationship (Fu et al., 2025; Pan et al., 2025).

### 1.5. Parent–Child Relationship and Child Mental Health and Well-Being

Understanding parent–child relationships during stressful events such as the COVID-19 pandemic is critical to understanding the influence on child mental and physical health. The style in which parents engage with their children can impact the child’s development of emotional regulation and engagement at home, school, and within the community (Deater-Deckard & Scarr, 1996). Children whose parents interact with them in a severe way tend to have more reported behavioral difficulties (Bates et al., 1995). While children whose parents communicate in a more open and understanding way tend to experience better emotional health (Ackard et al., 2006). Ultimately, the parent–child relationship is a possible focus that can be mediated to improve child health outcomes during a pandemic.

## 2. Materials and Methods

Data were collected from 571 caregivers (66% mothers, 32% fathers, 1.7% other caregivers) whose children ages ranged from 1 year to 18 years (*M* = 10.05; *SD* = 4.89). Respondents’ ages ranged from 18 to 72 years (*M* = 37.69; *SD* = 9.07). The participants were recruited online through Qualtrics during the Spring of 2023. Participants throughout the U.S. having at least one child whose age ranged from 1 to 18 years old completed an online survey and consent form. The study was approved by the Institutional Review Board at the authors’ institution.

In the survey, participants filled in items about demographic variables referring to the respondent (age, parental role, level of education, employment status, income) and their family (Where do you live with your family? What is your family’s income? Who is your child’s primary caregiver? Child age and gender). The survey also included questions for participants to identify their most difficult time period during the COVID-19 pandemic (When was the time you struggled the most during the COVID-19 outbreak and related restrictions? Why do you indicate this time as the time you struggled the most during the COVID-19 outbreak and related restrictions). The participants used the month and year they identified as a reference in answering the remaining survey questions regarding general anxiety (Spitzer et al., 2006), daily activity routine (Jensen et al., 1983), parent–child quality (Barnes & Olson, 1985), child social and mental well-being (R. Goodman, 1997), and child physical activity (Moore et al., 2020).

All key measures utilized a retrospective change scale. Participants first identified the specific month and year representing their personal lowest point during the pandemic (e.g., April 2020). All subsequent questions were framed relative to this period. For example: “*Compared to [Month/Year], when you were struggling the most, how would you describe the following today?*”

Items were rated on a 5-point scale: *Much less, A little less, About the same, A little more, Much more*. Responses were scored from 1 to 5, respectively. A score of 3 (“About the same”) served as the neutral baseline, representing no change from the worst period.

For positive constructs (e.g., daily routine quality, parent–child relationship, child well-being), scores above 3 indicated improvement, while scores below 3 indicated worsening. For the negative construct of parental anxiety, the scoring was reversed; a score below 3 indicated improvement (i.e., less anxiety), and a score above 3 indicated worsening. This scoring design established the neutral point (score of 3) as the direct statistical benchmark for assessing significant change either improvement or worsening from the participant’s self-defined pandemic crisis point.

The full list of items can be found in the Supplementary Materials. Table S1 indicates the measure adaptation overview.

## 3. Results

### 3.1. Data Analyses Plan

Preliminary analyses were conducted to examine the associations between demographic variables and the key study variables. Analyses of variance (ANOVAs) were used for categorical demographic variables (parental role, child gender, race/ethnicity, education level, income, employment status, and geographic region). To account for multiple comparisons and reduce the risk of Type I errors, a Bonferroni correction was applied, resulting in a per-test alpha level of 0.001. Pearson correlation analyses were performed for continuous demographic variables (parent age and child age) and the key study variables: parental daily routine, parental anxiety, parent–child relationship quality, and child mental and physical well-being.

To assess the degree of recovery from the peak of the pandemic, a series of one-sample *t*-tests were conducted. These tests determined whether participants’ current well-being scores significantly differed from a baseline value of 3, which represented “no change compared to their self-identified worst period during the COVID-19 pandemic.”

Third, to test the primary hypothesis that the parent–child relationship and parental anxiety mediate the link between routine disruptions and child outcomes, two separate simple mediation models (Model 4) were analyzed using the PROCESS macro for SPSS 25 (Hayes, 2017). One model predicted child mental well-being and the other predicted child physical well-being, with parental daily routine as the independent variable and both parent–child relationship and parental anxiety included as parallel mediators. Bootstrapping with 5000 samples was used to generate 95% confidence intervals for the indirect effects.

### 3.2. Descriptive Analyses

Bivariate analyses with nine demographic variables and five study variables resulted in 45 tests of statistical significance. With a desired overall alpha of 0.05, the Bonferroni correction resulted in an alpha level of 0.001 for each test (0.05/45 = 0.001). Based on a Bonferroni-corrected alpha level of 0.001, there were no significant associations between the key variables (parent daily routine, parent–child relationship, parent anxiety, child’s mental and physical well-being) and the demographics (parental role, child gender, race/ethnicity, educational level, income, employment status, and geographic region). Please see Supplementary Materials for each analysis.

Pearson correlation analyses were performed on the continuous demographic variables of parent age and child age, along with the key variables of the study, as detailed in Table 1. The analyses revealed a negative correlation between parental daily routine and parent age, suggesting that older parents reported less favorable daily routines. No significant correlations were found between either parent or child age and the child’s mental and physical well-being. In line with predictions, there were significant correlations between the child’s mental and physical well-being and parental factors, including parental daily routine, parental anxiety, and the quality of the parent–child relationship.

### 3.3. Improvements in Parent and Child Well-Being Compared to the Worst Period of the Pandemic

To assess the degree of recovery, we first tested whether reported well-being scores significantly differed from the baseline of ‘no change’ (a score of 3) compared to their self-identified worst period. Please see Table 2. Overall, participants indicated improvements across various dimensions including parent daily routine, parent anxiety, parent–child relationship, and the child’s mental and physical health, in comparison to the most challenging period of the pandemic. While these improvements were statistically significant, the magnitude of the changes was modest, with mean differences varying from 0.24 for child’s physical well-being to 0.62 for parent daily routine. Consequently, the data suggest that, although parents reported a relative improvement about themselves and their children from the pandemic, they had not yet returned to their pre-pandemic lifestyles.

### 3.4. Path Analysis of Parental Factors Impacting Child’s Mental and Physical Well-Being

Although we anticipated a direct relationship between improvements in parents’ daily routines and their children’s mental and physical well-being, this link turned out to be more complex, with parental anxiety and the quality of the parent–child relationship serving as mediation variables. To explore this mediated relationship, two separate Model 4 were employed using the PROCESS 3.1 Macro for SPSS (Hayes, 2017), each examining the mediation effect of two variables: parental anxiety and parent–child relationship. The data underwent a bootstrapping procedure with 5000 samples to generate 95% confidence intervals for the bounds of the effect sizes. Additionally, the index of mediation was used to assess the consistency of conditional indirect effects, with confidence intervals derived from bootstrapped standard errors (also based on 5000 samples). There was no multicollinearity among predictors: VIF ranged from 1.00 to 1.64 < 5, and Tolerance scores ranged from 0.61 and 0.99 > 0.1.

As hypothesized, a direct relationship was observed between the changes in parental daily routines and the mental well-being of their children (R2 = 0.12, b = 0.35, *p* < 0.01). Furthermore, parental daily routine showed a significant association with both the parent–child relationship (b = 0.62, *p* < 0.01) and parental anxiety (b = −0.09, *p* = 0.04). Additionally, the parent–child relationship (b = 0.38, *p* < 0.01) and parental anxiety (b = −0.36, *p* < 0.01) were significantly linked to the child’s mental well-being. The mediated effects are elaborated in Table 3. The analysis indicated that the parent–child relationship fully mediated the relationship between parental daily routine and the child’s mental well-being (R2 = 0.34, *p* < 0.01). The direct effect was no longer statistically significant (b = 0.08, *p* = 0.09).

As hypothesized, there is a direct relationship between the changes in parental daily routines and child’s physical well-being of their children (R2 = 0.27, b = 0.52, *p* < 0.01). Additionally, the parent–child relationship (b = 0.38, *p* < 0.01) and parental anxiety (b = −0.08, *p* = 0.02) were significantly related to the child’s physical well-being. The analysis indicated that the parent–child relationship partially mediated the relationship between parental daily routine and the child’s mental well-being (R2 = 0.36, *p* < 0.01) (Table 4). The direct effect remained statistically significant (b = 0.28, *p* < 0.01).

## 4. Discussion

The present study aimed to explore how the COVID-19 pandemic impacted parent–child relationships and, in turn, affected children’s social, behavioral, and physical well-being. The findings are consistent with prior research on the compounding stressors parents faced during COVID-19 and highlight the critical role of parent–child relationships in mitigating negative outcomes for children.

### 4.1. Parental Life Disruptions and Child Well-Being

Consistent with the literature (Lee et al., 2021), results indicate that disruptions to parents’ daily routines were significantly associated with poorer child outcomes. The strain of managing work-from-home duties, lack of childcare, financial insecurity, and restricted access to social support systems created an environment of heightened stress for parents (Aznar et al., 2021; Coyne et al., 2020; Singh et al., 2021b). Families were affected in different ways, but this variation was not linked to demographic differences, as suggested by the lack of significant associations between key variables and demographic factors. Demographic factors such as race, age, gender, and sexuality may shape individuals’ experiences of COVID-19-related stress, though findings remain inconsistent. Some studies report elevated stress among racial minorities, while others suggest the opposite (Luo et al., 2022; Montano & Acebes, 2020; Ponnock et al., 2021; Taylor et al., 2020b). Gender-based results also vary: while some research found no significant differences between men and women (Montano & Acebes, 2020; Szabo et al., 2020; Yan et al., 2021), others indicated that women were more vulnerable to pandemic-related mental health challenges (Ahuja et al., 2020; Mazza et al., 2020; Yalçın et al., 2021). Regarding marital status, multiple studies found no significant association with depression or anxiety during COVID-19 quarantine (Hawryluck et al., 2004; Nkire et al., 2021; Wang et al., 2020). Age-related findings suggest that individuals aged 41–50 may experience lower stress levels compared to younger adults (Balsamo & Carlucci, 2020; Huang & Zhao, 2020; Montano & Acebes, 2020; Rosi et al., 2021). Collectively, these findings align with our results, indicating that demographic differences often do not significantly influence COVID-19-related stress. Other factors driving the variation in effects across families remain to be discovered.

### 4.2. Parent–Child Relationship as a Central Mediator

A key contribution of this study is the demonstration of the mediating role the parent–child relationship plays between parental daily routine and child well-being. The mediation models support earlier research indicating that parental anxiety negatively affects parenting styles and the quality of parent–child interactions (Jackson & Choi, 2018; Ponnet et al., 2013). Here, the parent–child relationship fully mediated the association between parents’ daily routines and children’s mental well-being, and partially mediated the link with physical well-being. These findings emphasize that children’s psychological outcomes are particularly sensitive to relational quality within the household. This aligns with research suggesting that warm, communicative parenting styles foster emotional resilience in children (Ackard et al., 2006; Deater-Deckard & Scarr, 1996), while harsh or disengaged interactions are associated with increased behavioral problems (Bates et al., 1995; Low & Mounts, 2022). In this study, stronger parent–child relationships were associated with better child mental health outcomes, even when parents faced ongoing life challenges.

### 4.3. Parental Anxiety: A Partial but Weak Mediator

Parental anxiety was also tested as a potential mediator and, while significantly associated with child outcomes, its mediating effect was relatively small. This may be due to the complex and individualized nature of anxiety expression and coping styles, which are less observable and perhaps less directly disruptive to children compared to the quality of the parent–child relationship. Although previous studies have shown strong links between parental anxiety and child emotional problems (Barroso et al., 2018; S. H. Goodman et al., 2011), the current data suggest that the transmission of parental distress may more powerfully occur through disrupted parenting behaviors and strained parent–child dynamics.

### 4.4. Positive Trends but Incomplete Recovery

Encouragingly, participants reported modest improvements in parent–child relationships, daily routines, and both child mental and physical well-being from the worst period of the pandemic. These trends suggest resilience and partial adaptation over time. However, the small to moderate effect sizes and the fact that parents have not yet returned to pre-pandemic levels of functioning underscore the lingering effects of the pandemic. As Yuan et al. (2020) and Crawford (2021) suggest, the psychological impact of the pandemic may be prolonged, with continued support required for families.

### 4.5. Limitations

While informative, the study has several limitations. First, the use of self-report measures may introduce bias related to social desirability or retrospective recall, particularly when comparing to the worst time of the pandemic. Second, the cross-sectional nature of the data limits causal inference; though the path analysis supports hypothesized relationships, longitudinal data would better clarify directionality. Finally, the sample may not capture the most vulnerable populations, those without reliable internet access or those too overwhelmed to participate, potentially underestimating the true burden on some families.

### 4.6. Implications for Policy and Practice

The results underscore the importance of supporting parent–child relationships during large-scale societal disruptions. Intervention programs that target parenting communication, promote emotional warmth, and teach stress reduction and resilience techniques may serve as buffers against the negative impacts of crises like the COVID-19 pandemic. Moreover, policy efforts that reduce parental burden, such as flexible work schedules, child care support, and mental health services, can indirectly benefit children’s development by preserving healthy family dynamics.

## 5. Conclusions

This study contributes to a growing body of literature showing that the COVID-19 pandemic had wide-ranging effects on family systems, particularly through disruptions to daily routines and increased parental stress. The findings reaffirm that the parent–child relationship is a central mechanism through which parental well-being influences child development. Supporting parents in maintaining healthy routines and strong emotional connections with their children may be one of the most effective strategies to protect child well-being during future societal challenges.

## Figures and Tables

**Table 1:** Correlations for study variables.

Variable	1	2	3	4	5	6	7
1. Parent Daily Routine	—	−0.09 *	0.62 **	0.35 **	0.52 **	−0.09 *	−0.07
2. Parent Anxiety		—	−0.06	−0.39 **	−0.13 **	−0.08	0.01
3. Parent–Child Relationship			—	0.45 **	0.55 **	−0.02	−0.02
4. Child Mental Well-being				—	0.43 **	0.02	−0.03
5. Child Physical Well-being					—	−0.07	−0.03
6. Parent Age						—	0.53 *
7. Child Age							—

* *p* < 0.05; ** *p* < 0.01.

**Table 2:** Descriptive results.

Variable	Mean (SD)	Mean Differences	*t*	Cohen’s *d*
Parent daily routine	3.42 (0.96)	0.43	10.28 **	0.96
Parent–Child relationships	3.61 (0.90)	0.62	15.61 **	0.91
Parent anxiety	2.69 (0.89)	−0.31	−7.97 **	0.89
Child mental well-being	3.34 (0.58)	0.35	13.85 **	0.58
Child physical well-being	3.32 (0.59)	0.24	9.49 **	0.59

** *p* < 0.01.

**Table 3:** Mediation Model of Child’s Mental Well-being.

Child’s Mental Well-Being	*b*	*SE*	*LLCI*	*ULCI*	*t*	*p*
Constant			2.722	3.164	26.18	0.00
Parent’s Daily Routine	0.08	0.03	−0.0072	0.0998	1.70	0.09
Mediator: Parent Anxiety	0.03	0.02	−0.0005	0.0671	-	-
Mediator: P-C Relationship	0.24	0.04	0.1647	0.3176	-	-

**Table 4:** Mediation Model of Child’s Physical Well-being.

Child’s Mental Well-Being	*b*	*SE*	*LLCI*	*ULCI*	*t*	*p*
Constant			1.171	2.154	17.13	0.00
Parent’s Daily Routine	0.24	0.43	0.1697	0.3102	6.21	0.00
Mediator: Parent Anxiety	0.01	0.01	−0.0003	0.0203	-	-
Mediator: P-C Relationship	0.23	0.04	0.1638	0.3030	-	-

## Data Availability

Data available on request due to restrictions. The data presented in this study are available on request from the corresponding authors as open access data registration was not included in the Institutional Review Board application.

## Supplementary Materials

The following supporting information can be downloaded at https://www.mdpi.com/article/10.3390/bs15101423/s1, Table S1: Comprehensive Measure Adaptation Overview.

## Abbreviations

The following abbreviations are used in this manuscript:

P-C	Parent–Child
SD	Standard Deviation
