# Supplementary material for: Parental Stress, Parent-Child Relationship, and Child Wellbeing: A National Study of Family Life After COVID-19 Pandemic
Source: Behav Sci (Basel). 2025 Oct 20;15(10):1423. doi: 10.3390/bs15101423 (PMC12561007; doi:10.3390/bs15101423)
Supplement: Supplementary file 1 [file behavsci-15-01423-s001.zip › behavsci-3881575-supplementary.pdf]

Supplementary Material

Overview

Demographics (9 questions)

Pandemic questions (2 items)

Anxiety (7 questions)

Parenting Routine (10 questions)

P-C Quality (5 questions)

Child Wellbeing (15 questions)

Child Physical Activity (11 questions)

Demographics

1. **Select your birth month and years**
2. **What is your relation to the target child?**

    Mother

    Father

    Other

3. **Select the target child's birth month and year**
4. **Select the gender of the target child**

Male

Female

Transgender

Non-binary

Prefer not to say

5. **Are you a primary caregiver to this child?**

No

Yes

6. **Which of the following best describes your race and ethnicity? (Check all that apply)**

White

Black or African American

American Indian or Alaska Native

Hispanic or Latino

Asian

Native Hawaiian or Pacific Islander

Other ____

7. **What is your level of education?**

Completed middle school

Some high school

High school diploma

Some college

Associate's degree

Bachelor's Degree

Some graduate school

Professional degree (Masters or Doctorate)

8. **Which of the following describes your employment status over the course of the pandemic to now? (check all that apply)**

Student

Retired

Owning a business that still opens

Owning a business that has been shut down due to the pandemic

Working from home

Working away from home

Self-employed but out of work

Furloughed

Laid off

Taking a break from work

Full time

Part time

Other ____

9. Select your income category.

$0

$1 to $9 999

$10 000 to $24 999

$25 000 to 49 999

$50 000 to 74 999

$75 000 to 99 999

$100 000 to 149 999

$150 000 and greater

Prefer not to answer

Pandemic Question (2 items)

When was the time you struggled the most during the COVID-19 outbreak and related restrictions?

Why do you indicate ______ was the time you struggled the most during the COVID-19 outbreak and related restrictions? Please list all the reasons you struggled in the box blow:

General Anxiety (7 items)

Instructions:

Please answer the following questions and report on how your life is today compared to how it has been since ______ (month and year) - when was the time you struggled the most during the COVID-19 outbreak and related restrictions.

For example: Compared to _______ (month and year) when was the time you struggled the most:

Today, I am feeling ___ a little more__ nervous, anxious, or on edge.

Much less	A little less	About the same	A little more	Much more

Compared to _______ (month and year) :

1. Today, I am feeling ________ nervous, anxious, or on edge

2. Today, I am ______ being able to stop or control worrying

3. Today, I am worrying _____ about different things

4. Today, I am _______ trouble relaxing

5. Today, I am ______becoming easily annoyed or irritable.

6. Today, I am feeling _____ restless that it is hard to sit still

7. Today, I am feeling _______ afraid, as if something awful might happen

<u>Daily Activity Routines (10 items)</u>

Instructions:

Please answer the following questions and report on how your life is today compared to how it has been since ______ (month and year) - when was the time you struggled the most during the COVID-19 outbreak and related restrictions.

For example: Compared to _______ (month and year) when was the time you struggled the most:

Today, I have _a little more__ time for just talking daily with my children.

Much less	A little less	About the same	A little more	Much more

Compared to before the COVID-19 outbreak and related restrictions:

1. Today, I have ___ time for just talking daily with my children.

2. Today, I have ___ time for daily play with the children after coming home from work.

3. Today, I take care of the children ___.

4. Today, I read or tell stories to the children ___.

5. Today, my children do the same things each morning as soon as they wake up ___.

6. Today, my children take part in regular activities after school___.

7. Today, my children do their homework at the same time each day or night during the week___.

8. Today, my children go to bed at the same time almost every night ___.

9. Today, my family goes some place special together each week ____.

10. Today, my family has certain "family time" each week when they do things together at home __.

Parent–Child Relationship (5 items)

Instructions:

Please answer the following questions and report on how your life is today compared to how it has been since ______ (month and year) - when was the time you struggled the most during the COVID-19 outbreak and related restrictions.

For example: Compared to _______ (month and year) when was the time you struggled the most:

Today, I am _a little more__ satisfied with how my child and I talk together.

Much less	A little less	About the same	A little more	Much more

Compared to before the COVID-19 outbreak and related restrictions:

1. Today, I am ____ satisfied with how my child and I talk together.
2. Today, I show ____affection to my child.
3. Today, when I ask questions, I get honest answers from my child ___.
4. Today, I discuss problems with my child ____.
5. Today, I express all my true feelings to my child ___.

Child Social and Mental Well-being (15 items)

Instructions:

Please answer the following questions and report on how your life is today compared to how it has been since ______ (month and year) - when was the time you struggled the most during the COVID-19 outbreak and related restrictions.

For example: Compared to _______ (month and year) when was the time you struggled the most:

Today, my child considers other people's feelings __a little more___

Much less	A little less	About the same	A little more	Much more

Compared to before the COVID-19 outbreak and related restrictions:

1. Today, my child considers other people's feelings _____
2. Today, my child is _____ restless, overactive, cannot stay still for long
3. Today, my child shares ____ with other children, for example toys, treats, pencils, books, games, food
4. Today, my child loses temper ____
5. Today, my child prefers to play/be alone ___ than be with other youth
6. Today, my child is ____ well behaved, usually does what adults request
7. Today, my child worries ____
8. Today, my child is ____ unhappy, depressed or tearful
9. Today, my child is ____ distracted, concentration wanders
10. Today, my child is ____ nervous or clingy in new situations
11. Today, my child loses confidence ____
12. Today, my child is picked on or bullied by other children ____
13. Today, my child offers to help others (parents, teachers, other children) _____

14. Today, my child thinks things out before acting _____

15. Today, my child maintains good attention span, sees work through to the end ____

Physical Well-being (11 items)

Instructions:

Please answer the following questions and report on how your life is today compared to how it has been since ______ (month and year) - when was the time you struggled the most during the COVID-19 outbreak and related restrictions.

For example: Compared to _______ (month and year) when was the time you struggled the most:

Today, my child walks or bikes in the neighborhood __a little more___

Much less	A little less	About the same	A little more	Much more

Compared to before the COVID-19 outbreak and related restrictions:

1. Today, my child walks or bikes in the neighborhood ____.
2. Today, my child is doing physical activities or sport outside _____.
3. Today, my child is doing physical activities or sport inside _____.
4. Today, my child is doing household chores (e.g. cleaning, yard work)______.
5. Today, my child watches TV, movies, uses the computer for leisure _____.
6. Today, my child uses social media ______.
7. Today, my child does other sedentary leisure activities not in front of screens ____.
8. Today, my child sleeps ____.
9. Today, my child's sleep quality is ___.

10. Today, our family time spent in physical activity is ______.

11. Today, our family time spent in sedentary behaviors is _____.

Table S1: Comprehensive Measure Adaptation Overview

Construct	Citation	Items Adapted	Sample Adaptation
General Anxiety	GAD-7 (Spitzer et al., 2006)	7 items	Original: "Feeling nervous, anxious, or on edge" Adapted: "Today, I am feeling ______ nervous, anxious, or on edge compared to [worst period]"
Daily Routines	Family Routines Inventory (Jensen et al., 1983)	10 items	Original: "Time for just talking daily with children" Adapted: "Today, I have ______ time for just talking daily with my children compared to [worst period]"
Parent-Child Relationship	Parent-Adolescent Communication Scale (Barnes & Olson, 1985)	5 items	Original: "Satisfied with how we talk together" Adapted: "Today, I am ______ satisfied with how my child and I talk together compared to [worst period]"
Child Mental Well-being	Strengths and Difficulties Questionnaire (Goodman, 1997)	15 items	Original: "Considers others' feelings" Adapted: "Today, my child considers others' feelings ______ compared to [worst period]"

Construct	Citation	Items Adapted	Sample Adaptation
Child Physical Activity	Canadian 24-Hour Movement Guidelines (Moore et al., 2020)	11 items	Original: "Time spent in physical activity"<br>Adapted: "Today, my child does physical activities ______ compared to [worst period]"
